# Supplementary material for: Amino acid-dependent cMyc expression is essential for NK cell metabolic and functional responses in mice
Source: Nat Commun. 2018 Jun 14;9:2341. doi: 10.1038/s41467-018-04719-2 (PMC6002377; doi:10.1038/s41467-018-04719-2)
Supplement: Supplementary file 1 — Supplementary Information [file 41467_2018_4719_MOESM1_ESM.pdf]

Amino acid-dependent cMyc expression is essential for NK cell metabolic and functional responses in mice

Loftus et al.

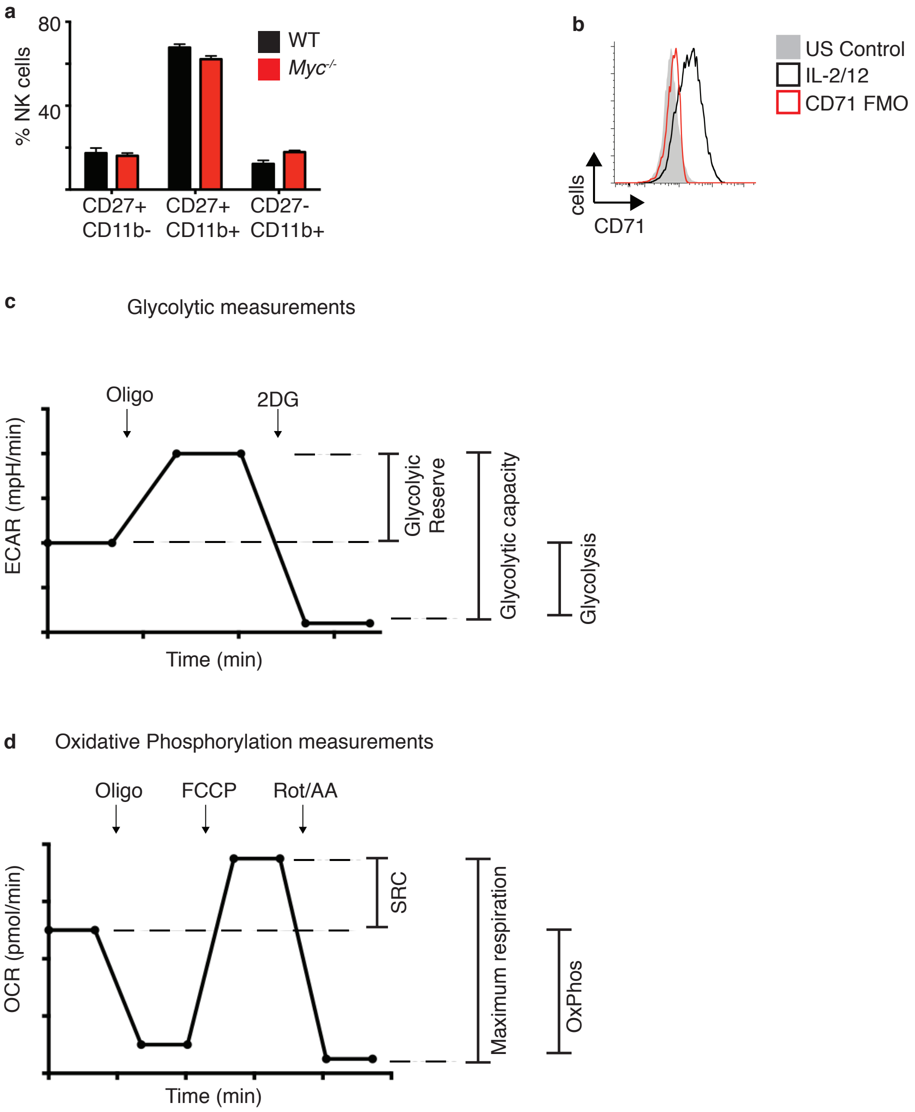

Supplementary Figure 1. Loss of cMyc does not affect the development or phenotype of unstimulated NK cells.
(a) Flow cytometry analysis of CD27/CD11b NK cell maturation marker expression on 6 day expanded WT and *Myc*$^{-/-}$ NK cells. **(b)** Flow cytometry analysis of CD71 expression on unstimulated or IL2/IL12 activated WT and *Myc*$^{-/-}$ NK cells. **(c-d)** Calculation of glycolytic reserve, glycolytic capacity and basal rate of glycolysis from extracellular acidification rate (ECAR) trace (**c**) and spare respiratory capacity (SRC), maximal respiration and rate of basal oxidative phosphorylation (OXPHOS) from oxygen consumption rate (OCR) trace (**d**). Data is mean +/- S.E.M of 3 (**a**) experiments, or representative of 3 (**b**) individual experiments.

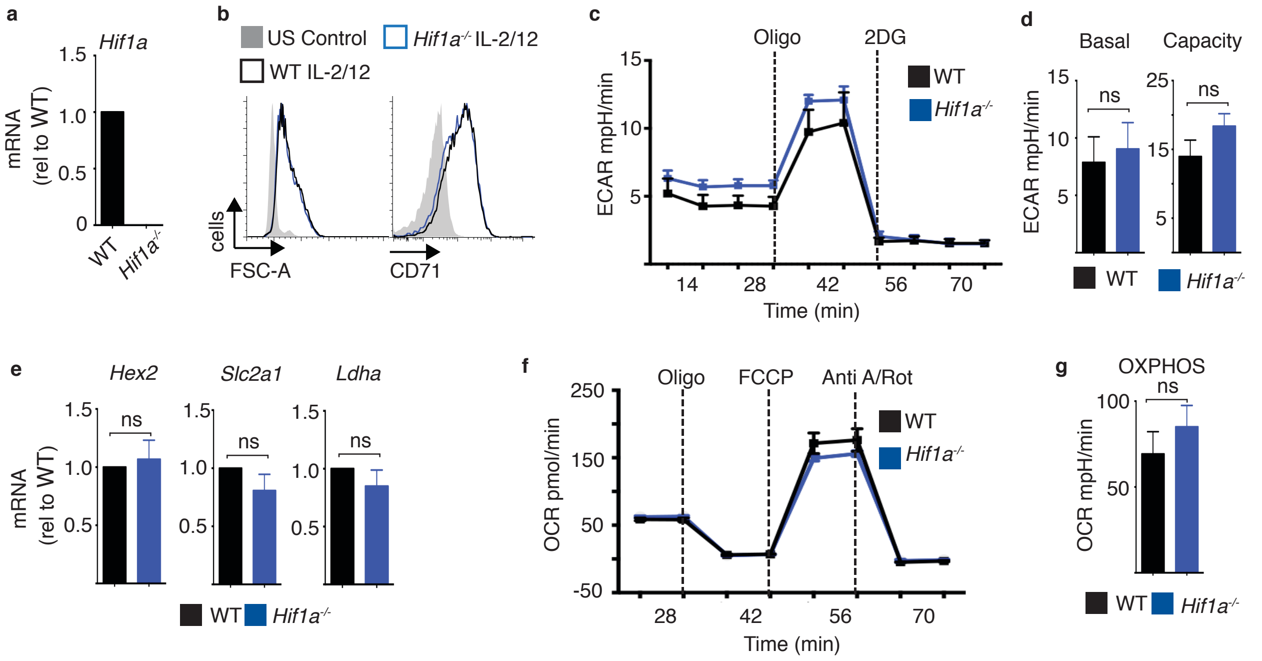

Supplementary Figure 2. HIF1α is not required for IL2/IL12 induced NK cell metabolism.

(a) 18 hour IL2/IL12 activated Hif1α$^{-/-}$ (Hif1α$^{flox/flox}$ x Vav-Cre) or WT (Hif1α$^{flox/flox}$) NK cells were analysed for *Hif1a* mRNA expression. **(b-g)** Hif1α$^{-/-}$ (Hif1α$^{flox/flox}$ x Vav-Cre) or WT (Hif1α$^{flox/flox}$) NK cells were left unstimulated or stimulated for 18 hours with IL2/12 before analysis. (**b**) Flow cytometry analysis for FSC-A and CD71 expression. (**c,d**) Analysis of NK cell extracellular acidification rate (ECAR) to assess basal glycolytic rate and glycolytic capacity. (**e**) qPCR analysis of mRNA for lactate dehydrogenase (*Ldha*), the Glut1 glucose transporter (*Slc2a1*) and hexokinase 2 (*Hex2*). **(f,g)** Analysis of NK cell oxygen consumption rate (OCR) to assess rates of OXPHOS and maximal respiration. Data is mean +/- S.E.M of 3 (**a**) or 4 (**d,e,g**) experiments, or representative of 4 (**b,c,f**) individual experiments. (**a, d, e, g**) Statistical analysis was performed using a student's t test (ns: not significant). Oligo, oligomycin; 2DG, 2-deoxyglucose; Anti A, antimycin A; Rot, rotenone; FCCP, Carbonyl cyanide-4-(trifluoromethoxy)phenylhydrazone.

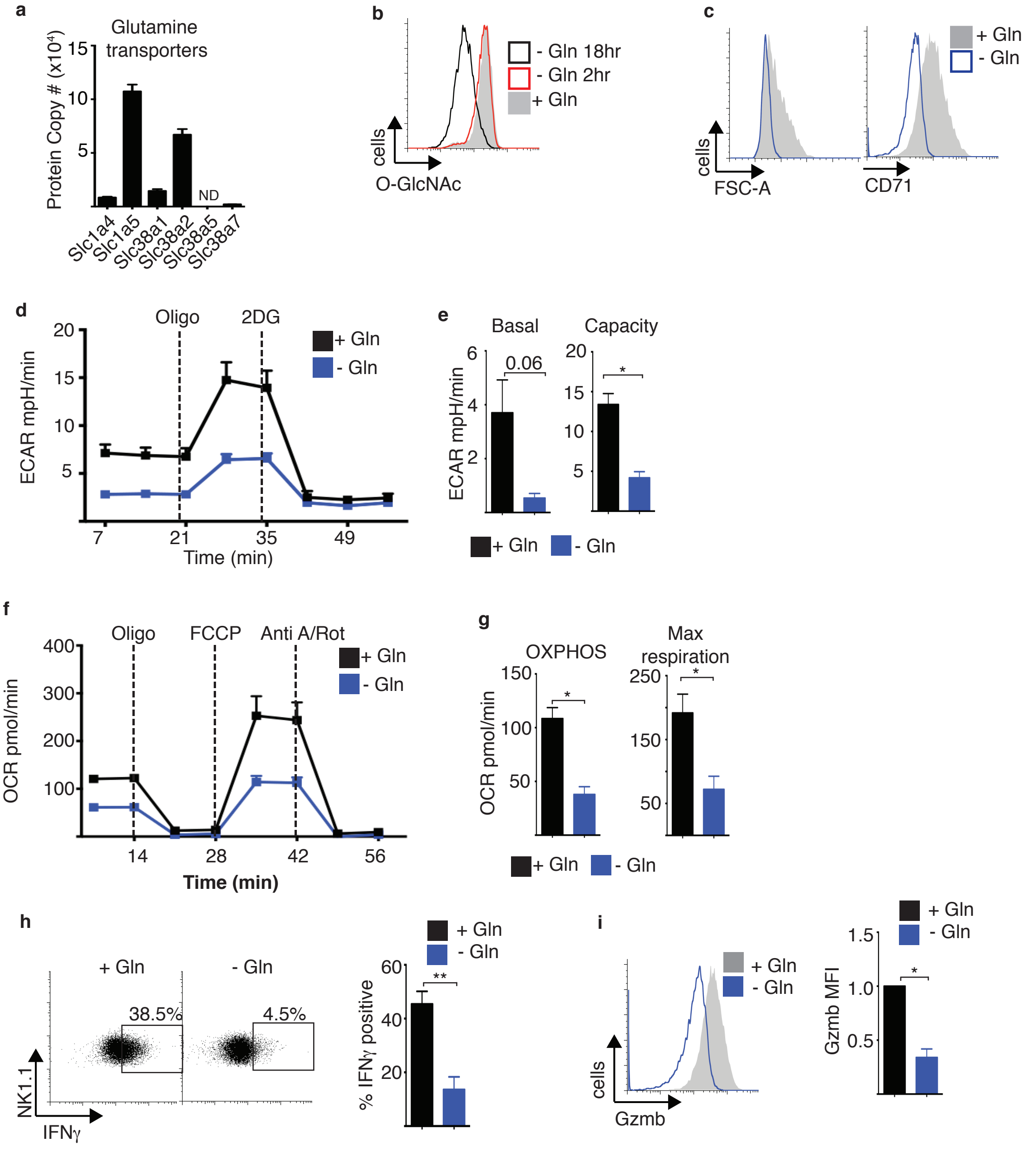

Supplementary Figure 3. Glutamine is required for NK cells metabolic and functional responses.
(a) NK cells were activated for 18 hours with IL2/IL12 and the number of protein copies per cell were determined for glutamine transporters SLC1A4, SLC1A5, SLC38A1, SLC38A2, SLC38A5 and SLC38A7. **(b)** 18-hour IL2/IL12 NK cells were cultured in the presence or absence of glutamine for different periods as indicated, before flow cytometry analysis for O-GlcNAcylation levels.
(c-i) NK cells were stimulated with IL2/IL12 in the presence or absence of glutamine for 18 hours as indicated. (**a**) FSC-A and CD71 expression were analysed by flow cytometry. (**b,c**) Analysis of NK cell extracellular acidification rate (ECAR) to assess basal glycolytic rate and glycolytic capacity. (**d,e**) Analysis of NK cell oxygen consumption rate (OCR) to assess rates of OXPHOS and maximal respiration. (**f, g**) IFNγ production and (**h, i**) granzyme B expression were analysed by flow cytometry. Data is mean +/- S.E.M of 3 (**c, e**) or 5 (**g, i**) experiments or representative of 3 (**b,d**) or 5 (**a,f,h**) individual experiments. Statistical analysis was performed using a students t test (**c,e,g**) or a one sample t test versus a theoretical value of 1 (**i**) (* p<0.05, **p<0.01). Oligo, oligomycin; 2DG, 2-deoxyglucose; Anti A, antimycin A; Rot, rotenone; FCCP, Carbonyl cyanide-4-(trifluoromethoxy) phenylhydrazone. ND, not detected.

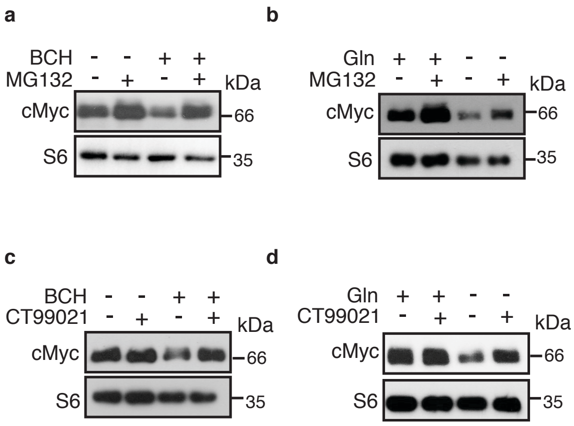

Supplementary Figure 4. cMyc levels are regulated by GSK3 mediated proteasomal degradation.
(a-d) NK cells were stimulated with IL2/IL12 for 18 hours and then in the presence or absence of BCH (25mM) (**a,c**) or glutamine (**b,d**) for 1 hour. The proteasomal inhibitor MG132 (3 μM) or the GSK3 inhibitor CT99021 (2 μM) was added for the final 1 hour where indicated. Samples were analysed by immunoblot to determine levels of cMyc and S6 ribosomal protein (S6). Data is representative of 3 individual experiments.

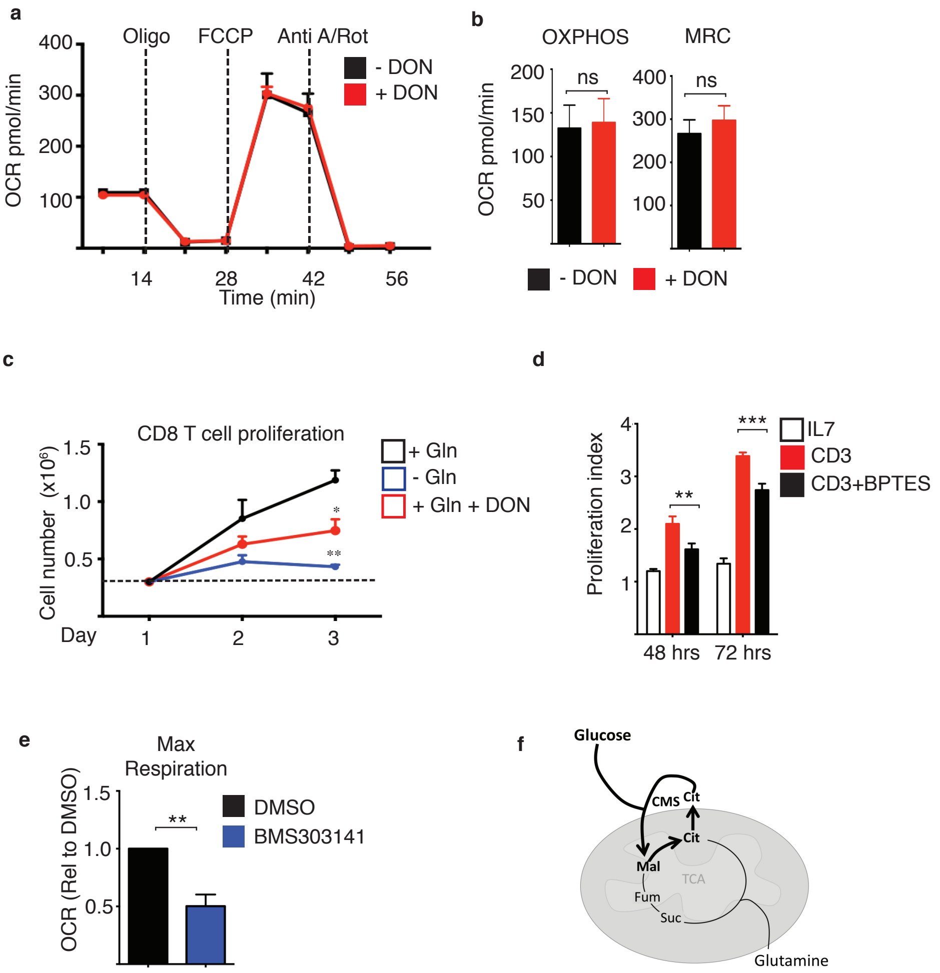

Supplementary Figure 5. Glutamine is an important fuel for T cell proliferation but does not fuel NK cell OxPhos.

(a,b) NK cells were stimulated with IL2/IL12 for 18 hours and then DON (2 μM) was added for 1 hour prior to seahorse analysis. Analysis of NK cell oxygen consumption rate (OCR) to assess rates of OXPHOS and maximal respiration. **(c)** IL2-induced proliferation of cytotoxic T lymphocytes (CTL) was measured as the increase in cell count after 48 hours (**c**) +/- glutamine, +/- DON. The dashed line indicates the density at which the cells were seeded. **(d)** Analysis of the proliferation index of CFSE stained activated $CD4^+$ T cells. **(e)** NK cells were stimulated with IL2/IL12 for 18 hours. The inhibitor BMS303141 or vehicle control were injected in the seahorse machine and OCR was measured for 1 hour after injection. **(f)** Schematic to show glucose derived citrate fuels the rate of NK cell OXPHOS via the citrate malate shuttle whereas anaplerosis is not an important fuel for NK cell OXPHOS. Statistical analysis was performed using a one sample t-test (**e**), a student's t test (**b**), a one way ANOVA with a Tukey test (**c**) or a two way ANOVA with a Tukey post hoc test (**d**). (* p<0.05, ns: not significant).

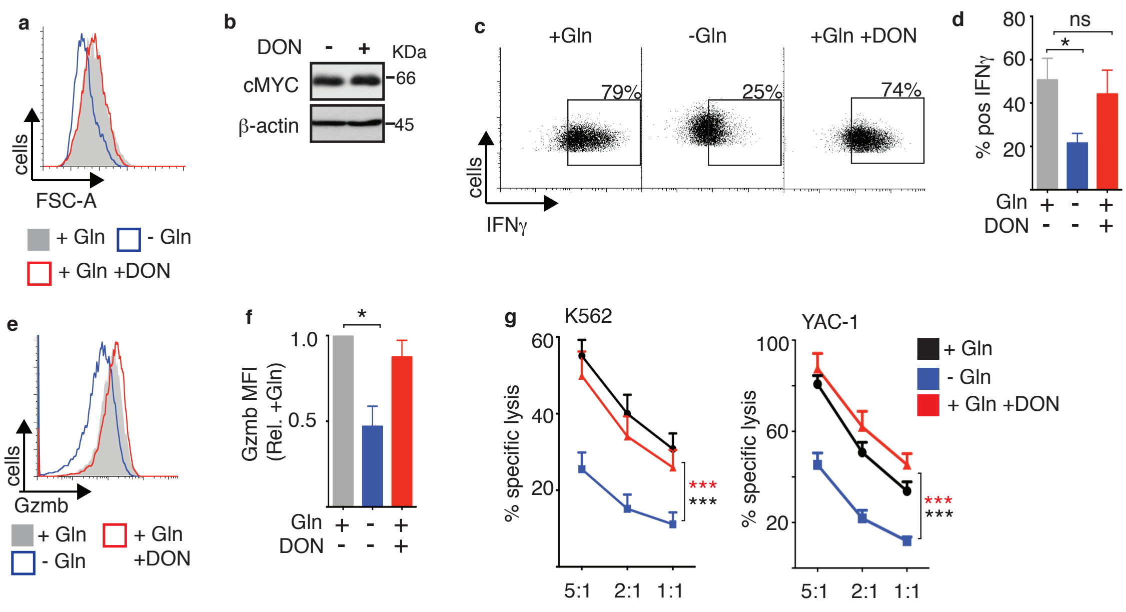

Supplementary Figure 6. Glutamine, but not glutamine metabolism, is required for NK cell anti-tumor responses. (a, c-f) NK cells were stimulated with IL2/IL12 for 20 hours and then switched into glutamine containing or depleted media in the presence or absence of DON (2 μM) for a further 20 hours before flow cytometry analysis of (**a**) FSC-A, (**c,d**) IFNγ or (**e, f**) granzyme B. **(b)** NK cells were stimulated with IL2/IL12 for 18 hours and then DON (2μM) was added for 1 hour prior to immunoblot analysis to determine the expression of cMyc and β-actin. **(g)** NK cell anti-tumor cytotoxicity was measured by stimulating NK cells with IL2/IL12 for 20 hours and then switching them into glutamine containing or depleted media in the presence or absence of DON (2μM) for a further 20 hours before co-culture with K562 or YAC1 tumour cells at the ratios indicated. Data is mean +/- S.E.M of 5 (**d, e,g**) experiments or representative of 4 (**b**) or 5 (**a,c,e**) individual experiments. Statistical analysis was performed using a one way ANOVA (**d,f**) or a two way ANOVA both with Tukey post-tests (**g**) (* $p<0.05$, ***$p<0.005$, ns: non-significant).

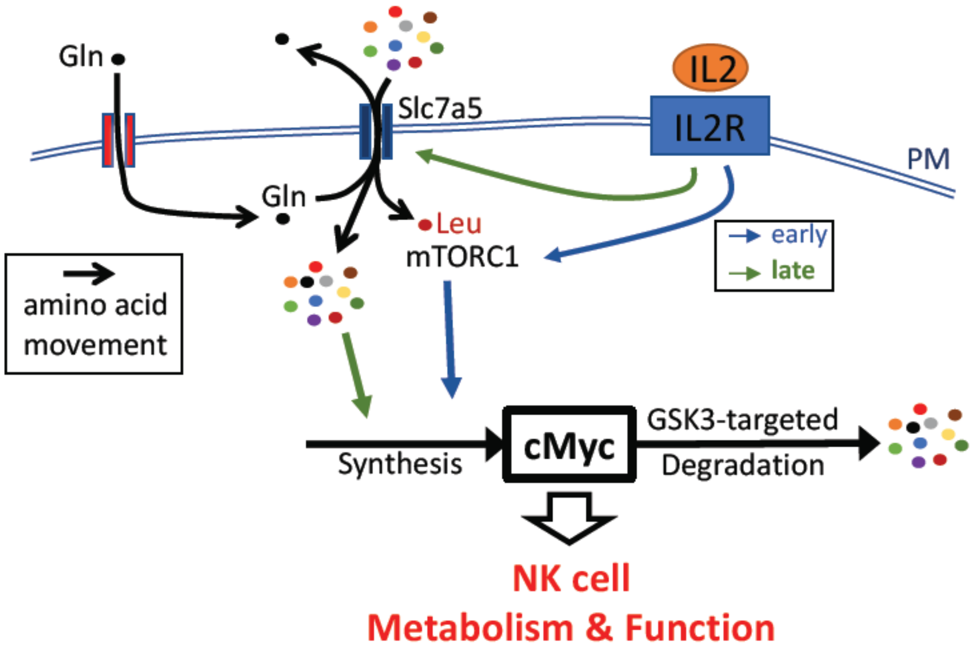

Supplementary Figure 7. Model for the regulation of cMyc expression in NK cells. cMyc levels are controlled by the balance of cMyc protein synthesis and GSK3-targeted degradation of cMyc in the proteasome. At early time points following IL2/IL12 stimulation (minutes) cMyc protein levels accumulated in an mTORC1 dependent manner. mTORC1 is activated by IL2 signalling and can increase cMyc protein translation through promoting 5' cap-dependent translation. IL2/IL12 stimulation also induces the expression and activity of the amino acid transporter SLC7A5. At later time points following IL2/IL12 stimulation (20 hours), amino acid uptake through SLC7A5 is essential to sustain cMyc protein levels while mTORC1 activity is not required. Withdrawal of glutamine, which is essential for SLC7A5 activity, or the direct inhibition of SLC7A5 mediated transport both result in rapid loss of cMyc protein expression. While leucine uptake through SLC7A5 is required for mTORC1 activity, at these later time points, leucine withdrawal is not sufficient to reduce cMyc expression. Therefore, the uptake of other amino acids through SLC7A5 are important in the regulation of cMyc, such as methionine, phenylalanine, tyrosine, arginine and tryptophan. Therefore, cMyc is a key regulator of NK cell metabolic and functional responses.

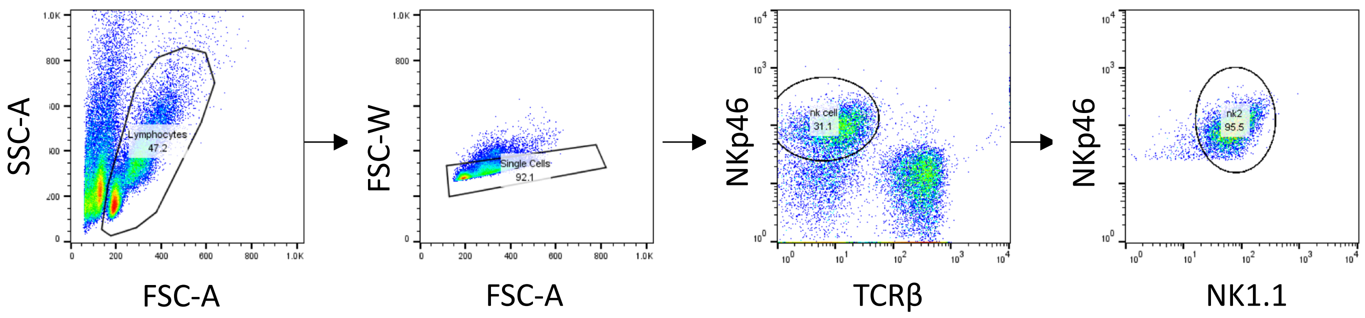

Supplementary Figure 8. Gating strategies for flow cytometry analysis.

Supplementary Figure 9- Uncropped Western blot images (page 1 of 3)

Figure 1a

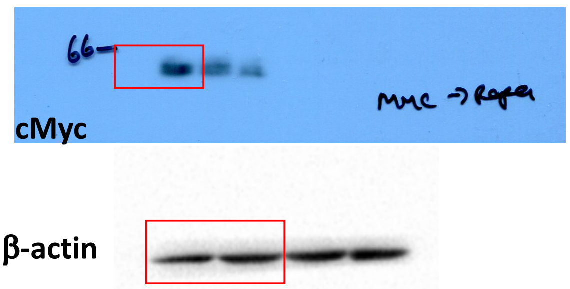

Figure 1b

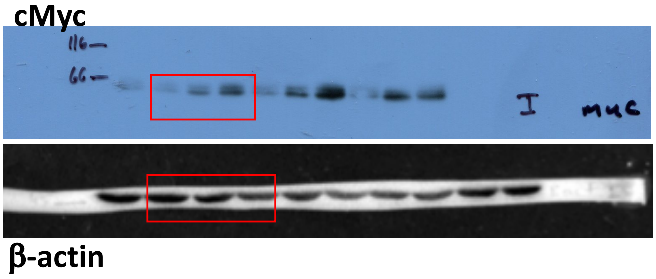

Figure 1c

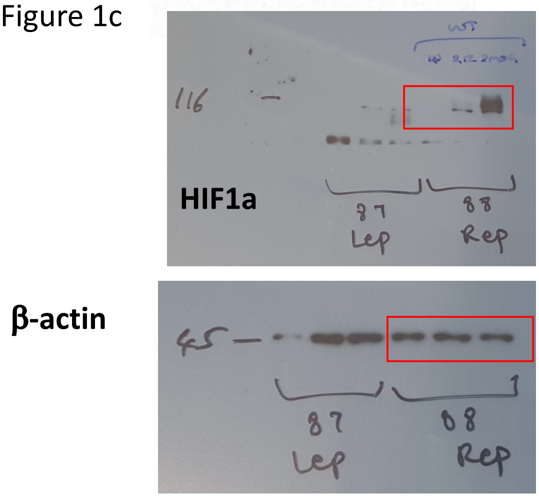

Figure 1e

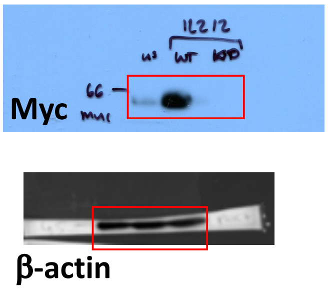

Figure 3a

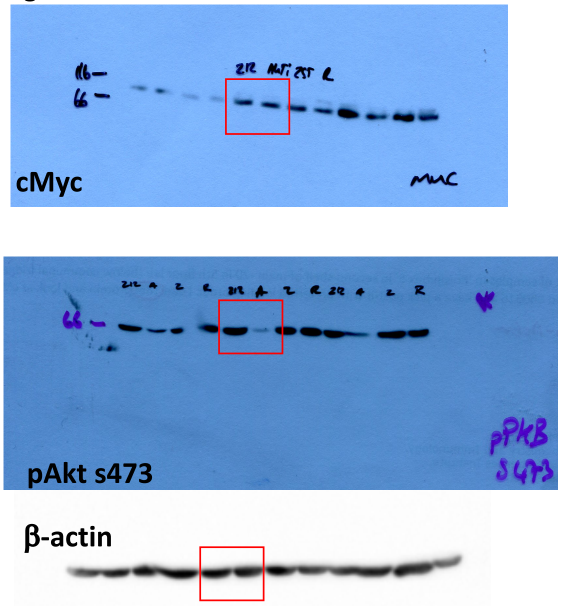

Figure 3h

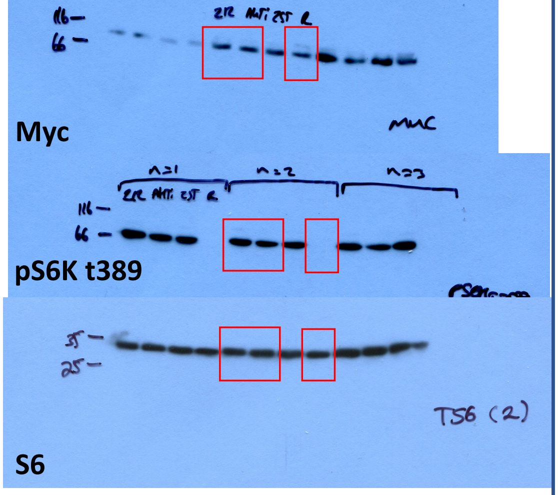

Figure 3i

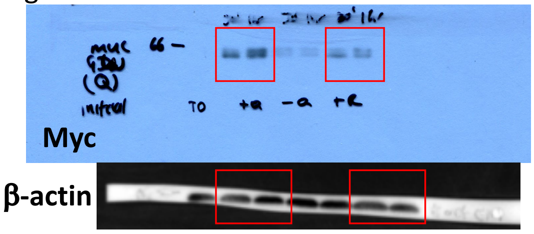

Figure 3b

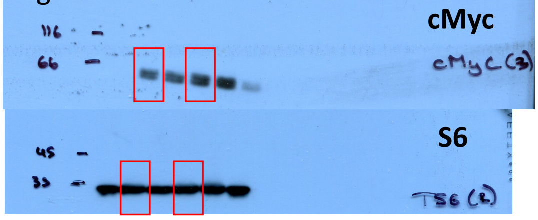

Figure 3j

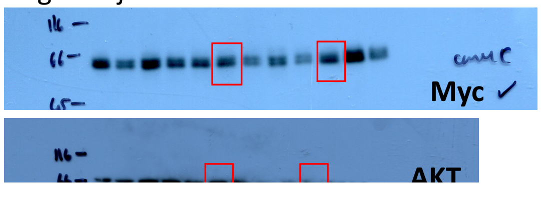

Supplementary Figure 9- Uncropped Western blot images (page 2 of 3)

Figure 3j

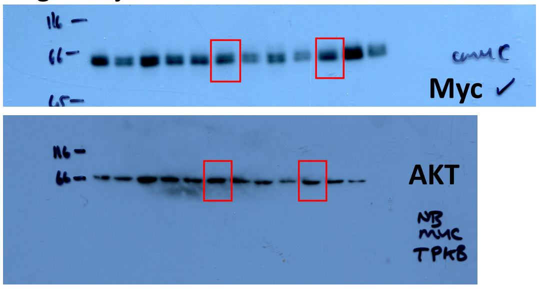

Figure 4e

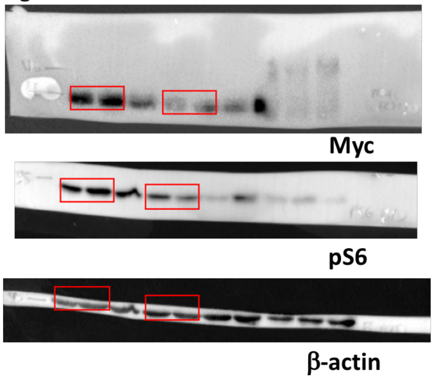

Figure 4f

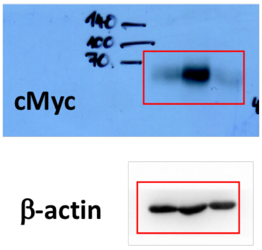

Figure 4g

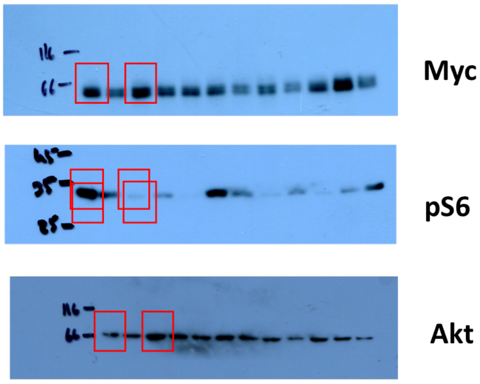

Figure 4i

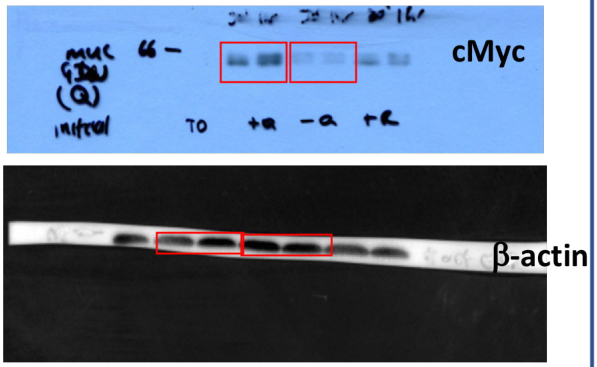

Figure 4i

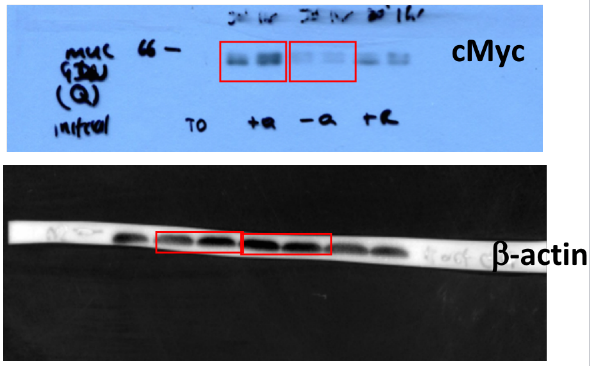

Figure 4j

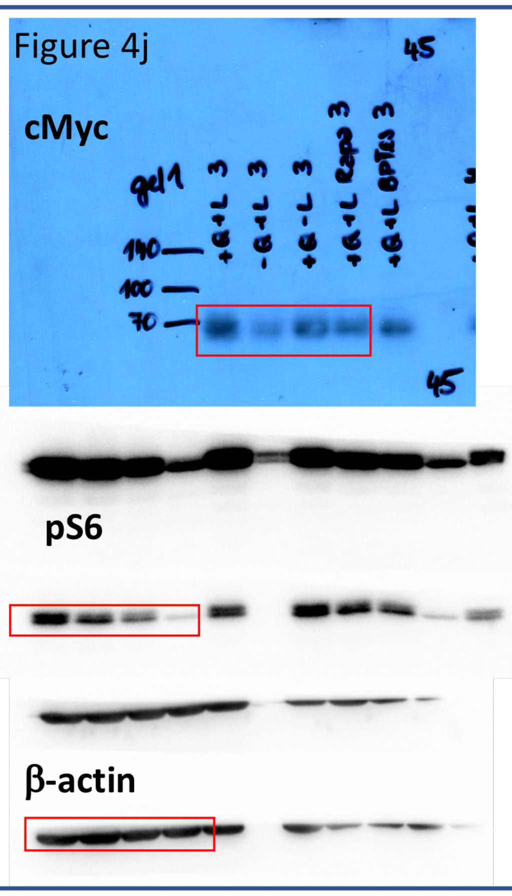

Supplementary Figure 9- Uncropped Western blot images (page 3 of 3)

Figure 8b

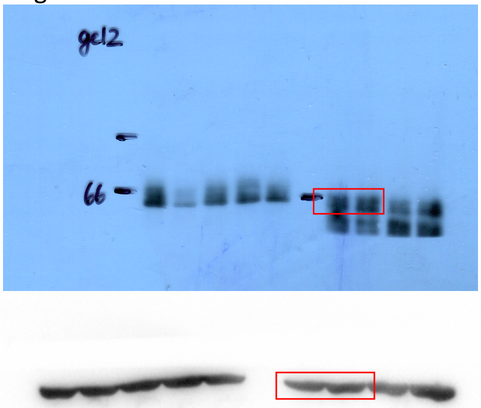

Supplementary Figure 4a

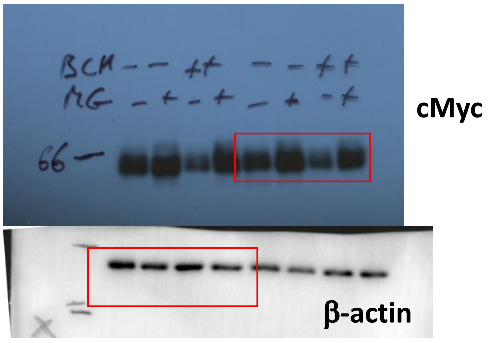

Supplementary Figure 4b

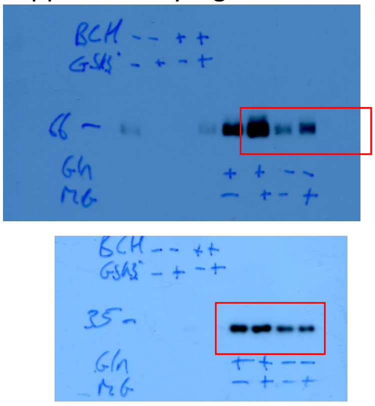

Supplementary Figure 4c

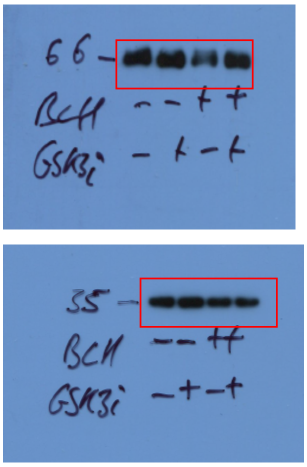

Supplementary Figure 4d

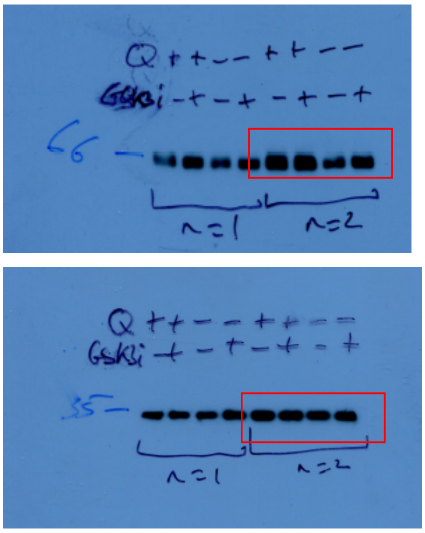

Supplementary Figure 6

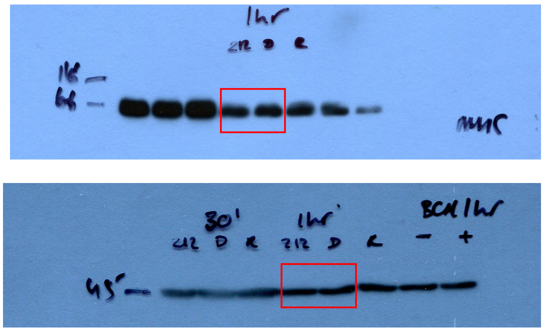

Primer	Direction	Sequence
**Rplp0**	Forward	5’-CATGTCGCTCCGAGGGAAG-3’,
**Rplp0**	Reverse	5’-CAGCAGCTGGCACCTTATTG-3’
**cMyc**	Forward	5’- GCGTTGGAAACCCCGACAG -3’
**cMyc**	Reverse	5’- CTTCCAGATATCCTCACTGGGC-3’
**Slc7a5**	Forward	5’- CTGGATCGAGCTGCTCATC-3’
**Slc7a5**	Reverse	5’- GTTCACAGCTGTGAGGAGC-3’
**Ldha**	Forward	5’-CTGGGAGAACATGGCGACTC-3’
**Ldha**	Reverse	5’-ATGGCCCAGGATGTGTAACC-3’
**Slc2a1**	Forward	5’-GGAATCGTCGTTGGCATCCT-3’
**Slc2a1**	Reverse	5’-CGAAGCTTCTTCAGCACACTC-3’
**Hex2**	Forward	5’-TCGCCTGCTTATTCACGGAG-3’
**Hex2**	Reverse	5’- CCATCCGGAGTTGACCTCAC-3’
**Phd3**	Forward	5’- CTGGTCCTGTACTGCGGGAG-3’
**Phd3**	Reverse	5’- GACCCCTCCGTGTAACTTGG-3’
**Tnf**	Forward	5’-AGGCACTCCCCCAAAAGATG-3’
**Tnf**	Reverse	5’-TGGTTTGTGAGTGTGAGGGTC-3’
**Pkm1**	Forward	5’-AAACAGCCAAGGGGGACTAC-3’
**Pkm1**	Reverse	5’- TTATAAGAGGCCTCCACGCTG-3’
**Pkm2**	Forward	5’- GCTATTCGAGGAACTCCGCC-3’
**Pkm2**	Reverse	5’- AAGGTACAGGCACTACACGC-3’

Supplementary Table 1. Primer sequences for rtPCR analysis.

Antibody target	Clone name	Source	Dilution
**cMyc**	D84C12	Cell Signalling Technologies	1:2,500
**phospho-S6 ribosomal protein$^{S235/6}$**	D57.2.2E	Cell Signalling Technologies	1:2,500
**phospho-S6K^{T389}**	1A5	Cell Signalling Technologies	1:5,000
**PKB**	C67E7	Cell Signalling Technologies	1:2,500
**β-actin**	AC-74	Sigma-Aldrich	1:10,000

Supplementary Table 2. Antibodies used for western blot analysis.
